# Examining the impact of total rewards on proactivity among Chinese knowledge employees: the moderating role of vertical and horizontal collectivist orientations

**DOI:** 10.3389/fpsyg.2025.1487464

**Published:** 2025-04-25

**Authors:** Jie Zhou, Junqing Yang, Bonoua Faye

**Affiliations:** ^1^School of Business Administration, Shanxi University of Finance and Economics, Taiyuan, China; ^2^School of Public Administration and Law, Northeast Agricultural University, Harbin, China

**Keywords:** total rewards, individual task proactivity, team member proactivity, vertical collectivism, horizontal collectivism, cultural orientations

## Abstract

**Introduction:**

In practice, the phenomenon of employees “lying flat”—characterized by poor proactive behavior—reflects a failure in the organization’s compensation incentive policy. Currently, the most effective compensation practice is total rewards, yet previous research seems to overlook the impact of total rewards (TR) on proactivity and has not considered when its effects may vary across different forms of proactive behavior.

**Methods:**

Based on social exchange theory and role theory, this study uses hierarchical regression and self-help methods to conduct a two-wave survey of the new generation of Chinese knowledge employees (*N* = 336).

**Results:**

The results show that total rewards significantly enhance both individual task proactivity and team member proactivity, with a greater positive effect on the former. Additionally, the study finds that vertical collectivism orientation weakens the total rewards-individual task proactivity relationship, while horizontal collectivism orientation weakens the total rewards-team member proactivity relationship. Surprisingly, the study does not support a positive moderating effect of vertical collectivism orientation on total rewards-team member proactivity or horizontal collectivism orientation on total rewards-individual task proactivity.

**Discussion:**

Our findings contribute to a deeper understanding of the effectiveness of compensation practices through the lens of intracultural heterogeneity and provide valuable insights for managers seeking to foster various forms of proactivity among the new generation of knowledge employees.

## 1 Introduction

Performance compensation, a key method for motivating employees, has gained significant attention from both scholars and managers (Chen et al., 2023). Furthermore, motivation is a perennial and fundamental topic within the management field, and it serves as one of the critical functions of economic managers. It embodies human resource management’s purpose and ultimate objective across organizations (Steel and König, 2006; Lee et al., 2021). Among the various formal Human resources management (HRM) motivational practices, compensation motivation is pivotal to organizational success. The trade-off between desired employee effort and employer compensation constitutes a core element of the employment relationship (Huang et al., 2018; Fulmer and Li, 2022). Compensation systems meticulously outline these exchanges’ details, components, and foundations. These systems define an organization’s relationship with its employees and influence workforce composition. By sending clear signals, they facilitate employee attraction, selection, and retention within an organization and guide their efforts throughout the workday (Gerhart and Rynes, 2003). As the global economy transitions into an era characterized by the convergence of the knowledge and digital economies, the primary component of the labor force is increasingly comprised of the new generation of knowledge-based employees (those born post-1990). Endowed with extensive knowledge and professional skills, these individuals are supplanting the traditional labor force as the principal contributors to human capital across all industries (Che et al., 2022). To accommodate the diversified job needs and preferences of employees, contemporary organizational compensation strategies have evolved beyond a focus solely on external economic rewards—such as salary, equity, and benefits—to include intrinsic non-economic rewards, as well as the integration of both internal and external compensation elements, collectively referred to as total rewards (TR) (Fulmer and Li, 2022). However, despite the widespread adoption of TR strategies among Chinese firms as a comprehensive incentive approach, the outcomes of these implementations have often been less than satisfactory (Jin and Yang, 2022). A recent survey by Hire.com found that over 80% of highly educated employees born after 1990 are content with the status quo, adopting a “lying flat” mentality. This raises important questions: why do strong compensation policies fail to motivate young intellectuals to be proactive, and what factors affect the effectiveness of these pay strategies?

Previous studies have explored the effectiveness of organizational reward policies, which can be broadly divided into two categories: economic compensation (e.g., salaries, performance bonuses, stock options) and non-economic rewards. The economic compensation category, especially pay for performance (PFP), has been widely studied from both economic and management perspectives. PFP is considered a critical mechanism for enhancing organizational efficiency, as it aligns employee performance with organizational goals (Huang, 2024; Park and Conroy, 2022). Economic theories like agency and tournament theory examine the loss of control and effectiveness of different PFP systems. From a management perspective, there is debate about how PFP influences creativity (Lin et al., 2022; Kong et al., 2023). The effectiveness of economic compensation is influenced by managerial roles, individual differences (e.g., culture, personality), demographics, group status, and policy clarity, affecting perceptions of PFP systems (Zhang et al., 2022). Non-economic rewards, such as leadership styles and work-life balance, impact employee attitudes and behavior, with key factors including cultural values, organizational climate, and leader-member exchange relationships (Nabella et al., 2022; Tano et al., 2023).

The effectiveness of organizational reward practices faces challenges due to the evolving nature of work and workforce composition, leading organizations to adopt TR systems that combine both economic and non-economic rewards (WorldatWork, 2024). Current research primarily examines individual compensation practices in isolation, with limited focus on the overall incentive effect of integrated compensation systems. This creates a gap between theoretical studies and real-world compensation practices (Fulmer and Li, 2022). Additionally, while Chinese knowledge workers’ reluctance to engage proactively (“lying flat”) is a key concern, theoretical research on organizational rewards and proactive behavior remains underexplored. Recent studies, such as by Mowbray et al. (2024), offer empirical evidence on the effectiveness of personal rewards tied to voice behavior. In reality, “lying flat” among employees takes various forms, including individuals who are unwilling to take the initiative and those who are open to personal change but lack the motivation to contribute to the team’s overall success (Strauss and Parker, 2015; Griffin et al., 2017). It is still necessary to further discuss which kind of proactivity is effective and which one is not.

Third, previous studies have shown that individual differences are the most important factors affecting the implementation of organizational reward policies (Fulmer and Shaw, 2018). In the existing studies on individual differences, no matter what kind of reward form, individualism/collectivism in cultural values is the most studied and influential dimension variable in the studies on individual differences. This conclusion has been confirmed by many scholars in the academic circle (Kirkman et al., 2017; Fan et al., 2022). Moreover, different cultural orientations can be activated in varying contexts, influencing corresponding psychological and behavioral outcomes (Huang et al., 2018; Gerhart and Rynes, 2003). China’s post-90s workforce, shaped by rapid economic growth and abundant resources, often consists of only children who possess qualities such as agility, self-efficacy, a love for challenges, and strong learning abilities. These distinctive traits are leveraged as they transition into knowledge-based roles, where they generate significant economic value and social impact (Tang et al., 2024). The collectivist values among this generation of young intellectuals seem to be undergoing a subtle evolution. They respect authority yet advocate for equality, demonstrating compliance and a drive for the initiative, embodying vertical and horizontal orientations (Zhang et al., 2022; Singelis et al., 1995). These cultural dispositions can coexist within the same context and situation or manifest independently within an individual. How do these two collectivist cultural tendencies affect the influence of organizational reward policies on their proactive behavior? Although assessing cultural differences to predict active behavior may be intuitively appealing, surprisingly little current research has examined the importance of vertical/horizontal collectivism to active behavior, and further research on this topic has been called for (Iqbal et al., 2024).

This study addresses gaps in compensation research by examining the effectiveness of TR policies in actual enterprise compensation practices, bridging theory and practice. It also tests the impact of compensation practices on various forms of proactive performance, offering insights into how different proactive behaviors are influenced by compensation. In this study, proactivity is divided into individual task proactivity (ITP), which involves the contribution of individual employees, and team member proactivity (TMP), which affects the whole team (Griffin et al., 2017; El Baroudi et al., 2019). Based on the social exchange theory, preferential treatment from organizations prompts employees to reciprocate with voluntary, exceeding behaviors (Ahmad et al., 2023; Jun and Eckardt, 2023). This paper addresses the additional gap in research on the relationship between reward and proactivity, exploring how different types of rewards influence various forms of proactivity. It also examines how vertical and horizontal collectivism, reflecting the cultural values of China’s new generation of knowledge employees, shape employees’ perceptions of organizational compensation. Social exchange theory highlights that while reciprocity is universal, individual responses vary due to factors like culture, social roles, and context, influencing attitudes and behaviors toward rewards (Ahmad et al., 2023; Tano et al., 2023; Zhang et al., 2022). Role theory posits that roles are flexible and subject to change, suggesting that individuals adapt their behavior based on their position within a role and their expectations from it (Anglin et al., 2022). Building on the cultural values framework proposed by Triandis and Gelfand (1998) and integrating social exchange theory and role theory, this study describes how different cultural orientations—such as vertical and horizontal collectivism influence the effectiveness of policies and behaviors in respective situations (Eagly and Wood, 2012). By addressing these questions, this research extends academic discussion on TR and varied proactive behaviors and analyzes why current compensation policies may fail. This exploration of intra-cultural heterogeneity offers practical insights for managers seeking to effectively engage the new generation of employees, thereby enhancing their potential and improving organizational efficiency.

## 2 Literature review and research hypothesis

### 2.1 Conception frameworks

#### 2.1.1 Individual task proactivity and team member proactivity

Proactive behavior represents a specific type of motivated behavior within workplace settings (Grant and Ashford, 2008). Originally, Bateman and Crant (1993) introduced this concept to organizational research, identifying it as a proactive personality—a relatively stable personality trait characterized by “a stable tendency to effect environmental change.” However, since Frese et al. (1996) introduced the concept of personal initiative, numerous scholars have examined proactive behavior as an individual-level behavioral tendency. This is a self-initiated, future-oriented action intended to effect change within oneself or the environment (Zhang Y. et al., 2023; Parker et al., 2006). Despite a growing body of research on proactive behavior, there remains a notable gap in the literature concerning classifying different proactive behaviors (Grant and Ashford, 2008; Belschak and Den Hartog, 2010; El Baroudi et al., 2019). Proactive behaviors come in various forms, including constructive, change-oriented, and feedback-seeking actions. These behaviors are expected to impact three main organizational levels: individual, team, and organizational. Some authors suggested that employees’ work behaviors are influenced by their social embeddedness within these entities (Griffin et al., 2017). Employees are more likely to act in the interests of their workgroup or organization when they see themselves as integral members. Individual task proactivity involves self-initiated, future-oriented behaviors aimed at improving personal work roles or skills. For example, a nurse might find a more efficient way to administer a drug. In contrast, when employees’ performance is linked to a team, they engage in actions that positively impact the team or organization, known as team or organizational member proactivity (Griffin et al., 2017). For example, employees might propose new approaches for their team, such as nurses suggesting improvements to shift schedules or offering ideas to enhance hospital policies (Williams et al., 2010).

Considering that frontline employees are often detached from the intricacies of organizational structure, systems, or workflow, they typically find it challenging to affect overarching organizational practices. In contrast, due to the interconnected nature of their tasks, communication and interaction within teams are frequent. Contemporary organizations increasingly value teamwork, and leaders progressively depend on individuals within these teams to address present and future challenges to enhance team performance (El Baroudi et al., 2019). Consequently, the primary focus of this paper is to differentiate between two categories of proactive behaviors: individual task proactivity and team member proactivity. Several points require clarification, particularly the distinction between these two types of proactivity. Individual task proactivity is focused on actions directed at oneself, while team member proactivity encompasses a broader scope involving contributions at the team level. The latter is inherently riskier as it requires navigating the complexities of team dynamics and systems (Cai et al., 2022). Furthermore, the distinction between team member proactivity and team proactivity needs clarification. Team member proactivity is an individual-level behavior, referring to independent, proactive actions taken by team members, remaining focused on individual contributions. In contrast, team proactivity reflects the collective ideas and efforts of the team as a whole, representing a team-level variable. However, this study does not address team proactivity at this time (Segarra-Ciprés et al., 2019; Emich et al., 2024).

#### 2.1.2 Total rewards

Technological advancements transform work dynamics as businesses shift toward a more virtual, knowledge- and service-oriented environment. Organizations are becoming flatter and more decentralized, with remote and complex work on the rise. Business managers increasingly recognize young, tech-savvy, and knowledgeable employees as critical drivers of productivity and performance (Ghlichlee and Motaghed Larijani, 2024; Cafaro, 2021; Gerhart and Rynes, 2003). The evolving business environment requires leaders to rethink how they attract and retain employees’ discretionary efforts. Traditional compensation elements like salary, benefits, and stock options are insufficient. Instead, TR has become a crucial strategy, addressing the diverse needs of the modern workforce while helping to manage rising personnel costs (Fulmer and Li, 2022).

TR is defined in two main ways. The narrower definition includes all compensation, benefits, and tangible elements like career development, often termed total compensation or remuneration. The broader definition provided by WorldatWork includes “everything an employee considers valuable in the employment relationship,” known as total value. In 2000, WorldatWork introduced a TR model with five components: compensation, benefits, work-life balance, performance and recognition, and development and career opportunities. It has since gained broader professional recognition globally (Cafaro, 2021; WorldatWork, 2024). The evolving nature of work, shifting skill requirements, and changing workforce expectations are transforming compensation into a critical organizational priority. Employers are increasingly focusing on TR, incorporating various elements such as healthcare benefits, retirement plans, flexible work options, professional development opportunities, and recognition programs. Greater emphasis is being placed on mental health, employee well-being, and personalized benefits tailored to individual needs and life circumstances. Additionally, organizations are striving to make compensation structures more equitable and transparent, aiming to attract and retain top talent, stay competitive, and succeed in a rapidly changing marketplace (Fulmer and Li, 2022; Xavier, 2014; Chiang and Birtch, 2012).

The TR model has proven to be a valuable and effective strategy for organizations across various industries and regions. Chinese companies are increasingly adopting this approach to design compensation systems and motivate employees. This growing interest has led Chinese scholars to explore TR, aligning its structure with Chinese cultural norms and workplace preferences and examining employee attitudes and behaviors toward it (Ji and Cui, 2021; Yan-qing and Wei-peng, 2019). Research on TR in China is still limited. Still, a notable study developed a comprehensive scale for measuring young Chinese employees’ perceptions of TR across dimensions like pay security, equity, workload, work experience, employee care, career development, and perceived personal value (Jin and Yang, 2022). In this study, it was used as a metric to assess TR.

#### 2.1.3 Vertical and horizontal collectivism

Culture is defined as a collection of conscious beliefs, norms, and values grounded in the morals, laws, customs, and practices of a society, typically at the national level. These are often referred to as holistic cultural values, with individualism–collectivism being one of the most commonly studied cultural dimensions (Hofstede, 1980). Due to the variation in individual values, extensive cross-cultural management research has been conducted at the individual level, examining how cultural value orientations influence societal actors, particularly in terms of their impact on the attitudes and behaviors of employees within organizations (Tano et al., 2023; Singelis et al., 1995; Spreitzer et al., 2005; Kirkman et al., 2017). As this study focuses on the new generation of knowledge employees in China, it specifically examines the collectivist cultural value orientation, which previous research has identified as being particularly prominent in the Chinese context (Cheng et al., 2016; Germani et al., 2021).

Triandis and Gelfand (1998) found that traditional cultural dichotomies do not adequately capture the complexity of cultural reality. Recognizing that individual differences in collectivism exist even within a single culture, they expanded the original dichotomy by introducing vertical and horizontal sub-dimensions, which reflect the relationship between power structures and collectivism. Specifically, vertical collectivism (VC) emphasizes hierarchical relationships within a group, where individuals prioritize group goals over personal interests, respect authority, and value social order. VC-oriented employees are motivated by competition with external groups and are more likely to conform to group norms and expectations, even at the cost of individual autonomy. In contrast, horizontal collectivism (HC) focuses on equality and interdependence within a group, where individuals view themselves as similar to others and prioritize harmonious relationships. HC-oriented employees value shared goals and avoid behaviors that disrupt group cohesion, often prioritizing interpersonal harmony over individual or competitive achievements. Importantly, the two dimensions are independent, not opposing ends of a continuum. Higher vertical collectivism does not imply lower horizontal collectivism (Singelis et al., 1995; Triandis and Singelis, 1998). Different cultural orientations may be activated in various environments, resulting in corresponding psychological and behavioral outcomes (Tano et al., 2023; Soh and Leong, 2002). Subsequent studies have demonstrated that both horizontal and vertical collectivism can directly influence various aspects of employee experiences, including perceptions, motivation (e.g., psychological needs), emotions (e.g., guilt and shame), emotional regulation, affect (e.g., subjective well-being), and behaviors (e.g., positive behaviors) (Hoxha and Ramadani, 2023; Germani et al., 2021; Ding et al., 2024), negative counterproductive behaviors (Suseno et al., 2021). At the same time, horizontal and vertical collectivism can serve as boundary conditions that influence behavior, such as the relationship between empowered leadership and employees’ psychological empowerment, as well as intrinsic motivation and creativity (Zhang et al., 2022; Thomas and Rahschulte, 2018).

Contemporary Chinese youth, particularly post-90s knowledge workers, exhibit vertical and horizontal collectivism. They also demonstrate a complex interplay of these orientations, transitioning from one tendency to another—such as a cognitive shift from authoritative to egalitarian perspectives, an attitudinal shift from obedience to initiative, and a behavioral shift from demanding to creating (Ding et al., 2024). In summary, the complex interplay of vertical and horizontal collectivism among contemporary Chinese youth, particularly post-1990s knowledge workers, reflects their ability to navigate both hierarchical and egalitarian perspectives. This dynamic results in varying psychological and behavioral outcomes depending on the context, highlighting the need to explore how different cultural orientations shape and modify employees’ psychological and behavioral responses.

In a nutshell, this study addresses key gaps in compensation literature by examining how total rewards (TR) influence proactive behaviors—individual task proactivity (ITP) and team member proactivity (TMP)—in a collectivist cultural context. Prior research lacks focus on proactive behaviors, treats collectivism as monolithic, and overlooks TR’s applicability in non-western settings. By differentiating VC and HC, the study reveals how cultural orientations moderate TR’s effectiveness. It bridges theory and practice, offering insights for tailoring compensation strategies in collectivist cultures, advancing social exchange and role theories, and enriching cross-cultural management research.

### 2.2 Research hypothesis

#### 2.2.1 Total rewards and different forms of proactive behavior

Social exchange theory helps explain the reciprocal dynamics influencing workplace behavior (Ahmad et al., 2023). The exchange process begins when an organizational actor either acts positively or negatively toward a target individual. In response, the target may exhibit two types of reactions. The first is a relational response, which either strengthens or weakens the social relationship with the actor, focusing on emotional exchange and openness. The second is a behavioral response, where the recipient engages in actions that either benefit or harm the actor, often more instrumental and exchange-driven. The resources exchanged in this reciprocity can be either tangible (e.g., goods, money, services, advice) or intangible (e.g., social recognition, respect, friendship, prestige) (Ahmad et al., 2023; Cropanzano et al., 2017). In addition to the fundamental principle of reciprocity, social exchange theory also encompasses other key principles, such as altruism (i.e., individuals may act to benefit others, even at high personal cost), competition (i.e., individuals may compete with out-groups to gain access to in-group resources), and group benefit (i.e., a rule that encourages contributions to the collective good when possible). Neglecting these principles or failing to recognize that multiple rules can operate simultaneously may lead to a distorted understanding of social exchange dynamics (Xiong et al., 2022; Almassaad et al., 2024).

Previous studies within the framework of social exchange theory have extensively explored positive reciprocal responses such as organizational citizenship behavior and extra-role behavior (Cropanzano et al., 2017). Scholars across various fields have demonstrated that initiative can yield numerous benefits. For individuals, task rewards can enhance core task performance, promote work engagement, boost both individual and organizational performance, and foster innovation and change. For teams, member rewards contribute to improved team effectiveness and overall performance (El Baroudi et al., 2019; Twemlow et al., 2023). However, proactive behaviors can also have negative effects on employees’ cognition, social relationships, and emotions. For example, deviating from established work routines can raise cognitive demands and incur additional costs (El Mansouri et al., 2024), leading to mental fatigue and reduced cognitive functioning. The uncertainty surrounding outcomes may induce anxiety (Cangiano et al., 2019), and such behaviors can also create friction with coworkers (Fan et al., 2022). This is primarily due to the nature of proactive behavior, which differs from routine actions. Proactive behavior extends beyond the formal responsibilities of a job role, involves experimenting with new or more effective methods, and carries inherent risks. It also requires ongoing improvement and innovation in task execution (Parker et al., 2006). This is particularly true for team member proactivity, as it involves not only individual actions but also the development of team structures, management of team workflows, and other aspects of team dynamics. As a result, it carries greater social costs compared to individual task proactivity (El Baroudi et al., 2019; Griffin et al., 2017). Therefore, there is a clear need for comprehensive, integrated, and systematic incentives to encourage this behavior effectively.

In alignment with the core tenets of social exchange theory, individuals engage in reciprocal exchanges, primarily aiming to secure long-term instrumental rewards and emotionally satisfy psychosocial needs (Klein et al., 2024). TR encompasses all the contextual resources accumulated through workplace roles, representing the overall value perceived by employees. These resources include economic rewards, such as salary, bonuses, equity, and promotions, which align with tangible resources in the reciprocal exchange. Additionally, TR includes non-economic incentives, such as increased time off, a focus on work-life balance, recognition, praise, and positive feedback, which correspond to the intangible resources in the reciprocal exchange (Cafaro, 2021; Fulmer and Li, 2022).

When financial and non-financial incentives work together—through the implementation of a comprehensive compensation policy—they address the diverse needs of employees. This, in turn, enhances proactive self-efficacy, fostering greater enthusiasm, focus on tasks, and increased effort. As a result, employees are more likely to persist and feel empowered to mobilize all of their resources to achieve their goals, even in the face of challenges and uncertainty (Grant and Ashford, 2008; Ma et al., 2020). Employees are motivated not only to complete their assigned tasks but also to think creatively about improving their work processes. While this may involve cognitive and social costs, the rewards help protect their resources and values. As a result, employees are driven to bridge the gap between their current situation and the desired outcome, demonstrating individual task proactivity in the process (Strauss and Parker, 2015).

At the same time, total rewards can provide employees with a sense of psychological security and organizational identity, encouraging them to act out of initiative because they “can do” and “reason to do.” This not only drives individual task initiative but also motivates them, as team members, to implement changes that benefit the entire team (Liu et al., 2017). Research has shown that a mixed approach to rewards is more effective in eliciting positive emotions and energy. This approach helps employees overcome self-doubt and self-depletion, which can arise from uncertainty about the outcomes of their actions, and encourages them to actively engage in team interactions (El Baroudi et al., 2019). Drawing on the principles of altruism, competition, and group benefits within social exchange theory, TR—as an integrated compensation system—can foster pro-social motivation. This reminds employees of the significance of their roles as members of a larger organization, such as a team, and reinforces their sense of contribution to the collective good (Ahmad et al., 2023). The desire to improve the efficiency and benefits of the group through individual contributions motivates individuals to engage in proactive behaviors aimed at enhancing teamwork. Research has also shown that organizations can bridge the gap between the current situation and long-term goals by implementing vision-centered interventions. Such interventions stimulate team member proactivity by encouraging individuals to think beyond their current roles and fostering interest in driving broader changes within the group and organization (Fay et al., 2023).

However, as mentioned earlier, team member proactivity involves a broader focus on team-level changes and requires maintaining harmony and interaction with others. As a result, they are inherently more uncertain than individual task proactivity. The higher the uncertainty, the greater its impact on the degree of formalization in the job. This causes employees to carefully weigh their potential behaviors and the associated risks before acting, leading to increased anxiety and defensiveness. As a result, it may trigger withdrawal behaviors (Liu et al., 2017). Thus, the recognition, encouragement, and resources provided through total rewards act as supportive signals from the organization, reinforcing employees’ psychological contracts and encouraging positive behaviors while reducing concerns about risk. However, the risk-reducing effect is likely to be more pronounced for individuals than for team members. This is particularly true for young knowledge workers in China, who prioritize self-expression and have a strong need for organizational recognition and support, along with a focus on personal and professional development. Team member proactivity, which requires more extensive interaction and alignment with others, may not align as well with these individual priorities. Based on these considerations, the following hypotheses are proposed in this study:

Hypothesis 1 (H1):

*H1a*: Total rewards may be positively associated with individual task proactivity (ITP).

*H1b*: Total rewards may be positively associated with team member proactivity (TMP).

*H1c*: The positive effect of total rewards on individual task proactivity is stronger than its effect on team member proactivity.

#### 2.2.2 The moderating role of vertical and horizontal collectivist cultural orientations

Social exchange theory acknowledges that while reciprocity is a universal exchange norm between individuals, it does not imply that all individuals value reciprocity to the same extent. Significant cultural and individual differences primarily influence the specific patterns of reciprocity (Ahmad et al., 2023; Chen et al., 2009). There is substantial evidence supporting this view, such as the finding that individual differences in future orientation positively moderate the relationship between vision-centered interventions and the development of proactive skills, as well as between proactive skills and organizational members’ initiative (Strauss and Parker, 2015). This study focuses on the boundary conditions of differences in employees’ collectivist cultural orientations because its subjects are China’s new generation of knowledge workers. Research has shown that a distinctive characteristic of this group is the complex intersection of horizontal and vertical collectivist values (Ding et al., 2024).

Moreover, existing literature has validated the effectiveness of different collectivist orientations on various organizational practices, such as leadership behaviors (Fan et al., 2022), PFP (Zhang et al., 2022), LMX (Tano et al., 2023). Role theory (RT) offers a passive explanation for variations in reciprocity, suggesting that differences in outcomes may arise from an individual’s social position and role. From a social-psychological perspective, RT emphasizes how these roles and statuses, within structures like organizations, define behavioral norms and expectations, influencing individual actions (Eagly and Wood, 2012; Anglin et al., 2022). Different cultural orientations shape individuals’ attitudes and motivations toward organizations, groups, and others (such as leaders and colleagues). Cultural orientations influence how individuals perceive their connection to society and others. Employees with different horizontal and vertical collectivist orientations, for example, are expected to exhibit distinct traits and behavioral norms. This is because their identification with specific internal groups shapes their self-concept. As a result, their roles within these groups lead to varying interpretations of responsibilities and obligations, both toward the group and toward individuals, affecting their attitudes and behaviors in organizational and social contexts (Rotundo and Xie, 2008; Hofstede, 2011).

Individuals who embrace vertical collectivism define themselves as part of a collective, adhering to group norms, respecting authority, and willingly sacrificing their personal interests for the benefit of the in-group (Singelis et al., 1995). In other words, vertical collectivists view the group as a source of their responsibilities and obligations. They inherently believe that building a strong and cohesive in-group is their primary goal. To achieve this, they strive to ensure their group surpasses others, and by doing so, they demonstrate unwavering loyalty to their role within the group (Hofstede, 2011). Such individuals are inherently prosocial, exhibiting a strong sense of collective responsibility and a shared destiny. Therefore, when employees with a high orientation toward vertical collectivism join a work team, they emphasize their membership within the team, strongly identify with it, and internalize its rules to align with the team’s expectations (Anglin et al., 2022). They actively strive for their team to outperform others (Lee et al., 2021).

Moreover, team members’ proactivity, compared to individual task initiative, is primarily aimed at enhancing the overall performance of the team and improving its operational efficiency, which is inherently prosocial (El Baroudi et al., 2019). Based on social exchange theory, when an organization provides comprehensive rewards that meet diverse employee needs, employees with a high orientation toward vertical collectivism naturally perceive these signals of support and rewards as the organization’s way of enhancing their group’s competitiveness and success. This perception strengthens their motivation for reciprocal exchange and their sense of obligation to give back, reinforcing their belief in mutual reciprocity. Consequently, they are more likely to exert extra effort, increasing the likelihood of team-oriented actions and fostering team member proactivity (Chen et al., 2009).

In contrast, individual task proactivity is more oriented toward personal achievement and does not involve collaboration with colleagues or the collective. It primarily focuses on individual benefits, such as career success and performance improvement. Employees with lower vertical collectivism tend to prioritize personal effort over collective effort, emphasizing their individual gains, losses, and accomplishments. Furthermore, such employees are less inclined to uphold authority and hierarchical norms, making them more likely to challenge these structures and engage in innovation. Their instrumental focus on economic outcomes, driven by the motivation for reciprocity in rewards, may lead them to adjust their task plans to optimize personal benefits (Triandis and Gelfand, 1998; Triandis, 2001). The above explanations provide evidence to infer that when leaders call for a sense of obligation to pursue a shared vision, employees with high vertical collectivism experience a stronger sense of purpose. They inherently feel a duty toward the collective, which inspires greater creativity (Fan et al., 2022). Studies have also shown that employees with high vertical collectivism are more supportive of hierarchical structures and resource allocation based on fairness (Zhang W. et al., 2023; Hoxha and Ramadani, 2023).

As outlined above, from a social exchange perspective, vertical collectivist employees’ beneficial behaviors reflect active reciprocity, while role theory views them as fulfilling passive obligations (Eagly and Wood, 2012). Strong vertical collectivism drives adherence to norms and contributions to collective interests as employees fulfill their expected group roles and duties. For employees with strong vertical collectivism, maintaining collective interests is a key role responsibility. They are also more likely to be expected by others (e.g., leaders and colleagues) to engage in behaviors that strengthen group cohesion and contribute to collective benefits (Zhang W. et al., 2023; Moon et al., 2023). Chinese collectivists are less prone to social loafing due to social motivations and fear of losing “face.” Organizational rewards and expectations reinforce their commitment to role-consistent, team-benefiting behaviors. Employees with high vertical collectivism are aware of external and internal expectations, prioritize collective goals over personal interests, and reinforce the commitment to team-benefiting behaviors (Earley, 1989). However, when individual and organizational goals conflict, this can create role conflict, particularly for those driven by personal task proactivity (Anglin et al., 2022). Employees with lower collectivism, focused on individual goals, are more easily understood by the organization. This leads to the formulation of the following hypothesis:

Hypothesis 2 (H2):

*H2a*: Vertical collectivism (VC) weakens the positive relationship between total rewards (TR) and individual task proactivity (ITP).

*H2b*: Vertical collectivism (VC) strengthens the positive relationship between total rewards (TR) and team member proactivity (TMP).

Individuals with horizontal collectivism are not oriented toward the collective as a whole but rather toward the members within the group. They define themselves in terms of interdependence with other group members, viewing themselves as equals and not mindlessly pursuing respect for authority. They place a high value on social harmony and focus on maintaining social bonds with others within an egalitarian framework (Singelis et al., 1995). Individuals with this characteristic prioritize teamwork and work to maintain emotional stability and cohesion within the team. They do not place their group’s interests above those of other groups. Instead, their main focus is on maintaining harmonious interpersonal relationships and fostering friendly ties within the team (Triandis and Gelfand, 1998; Fan et al., 2022).

Therefore, despite having a strong sense of team spirit, employees with a high orientation toward horizontal collectivism exhibit minimal competitive drive and a lower desire to alter team operations to improve overall efficiency and performance proactively. This is because team proactivity is considered a risky behavior that comes with significant costs. Research has shown that the primary costs involved are social relationship costs, such as receiving lower performance evaluations from leaders, threatening leadership, and provoking jealousy or exclusion from colleagues. These factors contribute to the “dark side” of proactivity research, highlighting the social risks associated with taking the initiative (Parker et al., 2019; Twemlow et al., 2023). This is primarily because as the interdependence of tasks within a team increases, any change in how one member performs their tasks can impact the tasks and workflows of others, potentially disrupting established team norms (El Baroudi et al., 2019). However, colleagues may not be receptive to adapting to new approaches or abandoning their previous habits, which can lead to dissatisfaction or even resentment from both leaders and coworkers.

Based on social exchange theory, team members with a strong orientation toward horizontal collectivism carefully weigh the benefits and costs of maintaining and strengthening positive interactions within the group. They compare the TR provided by the organization with the potential social relationship costs that might arise. Given that employees with high horizontal collectivism prioritize the feelings of other group members, tend to cooperate, and adhere to established norms, this is likely to diminish their proactivity within the team. Similarly, from the perspective of role theory, horizontal collectivism represents a social framework in which an individual’s role identity is rooted in their connection to the broader social collective. This model relies on continuous interdependence and alignment with others (Iqbal et al., 2024). They weigh the benefits and costs, prioritize the feelings of others, and strive to maintain and enhance positive interactions within the group, embodying the attributes of altruism in pro-social behavior (Moon et al., 2018). When the organization provides recognition, it is expected that employees will engage in behaviors that align with their characteristics and help maintain team harmony. This expectation is supported by relevant research, which suggests that employees with high collectivism are more likely to balance their sense of duty to pursue the organization’s vision with the need to maintain harmony within the group. Furthermore, studies have found that individuals with high collectivism tend to prioritize ensuring equality and prefer collaborating with internal groups that align with social expectations and organizational norms, thereby developing a moral identity that conforms to these standards (Fan et al., 2022; Smithikrai, 2014).

However, when employees with a horizontal collectivism orientation work for their own interests rather than for the team, they are less concerned with the needs of other members. In this context, traits such as the pursuit of equality between individuals and the emphasis on equal expression of individual rights become more prominent. These employees are less focused on top-down hierarchical relationships, instead emphasizing individual agency and autonomy. They are less likely to follow authority, which fosters self-identity and self-expression, enabling them to share their innovative and transformative ideas confidently and unthinkingly. When organizations offer a variety of rewards, both economic and social, their willingness to reciprocate is strengthened, and their role identity as independent, equal, and free is affirmed. This, in turn, significantly enhances their proactivity in personal work environments and methods (Klein et al., 2024). Previous research has reinforced this idea, suggesting that individuals with high horizontal collectivism (HC) who value equality within the group are more willing to express concerns about any injustices or inappropriate behaviors to ensure everyone is treated equally. It is generally believed that horizontal collectivism strengthens the positive relationship between psychological empowerment and proactive behavior (Seibert et al., 2011; Iqbal et al., 2024). Based on these observations, this paper proposes the following hypothesis:

Hypothesis 3 (H3):

*H3a*: Horizontal collectivism (HC) enhances the positive relationship between total rewards (TR) and individual task proactivity (ITP).

*H3b*: Horizontal collectivism (HC) weakens the positive relationship between total rewards (TR) and team member proactivity (TMP).

**Figure 1:**
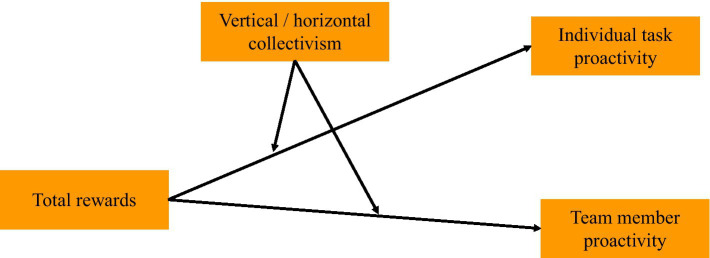
The conceptual model of this study.

In a nutshell, this paper builds the research model shown in Figure 1.

## 3 Method

### 3.1 Study sample and data collection

Questionnaires, field interviews, and social surveys are commonly used tools in empirical social science research (Faye et al., 2024; Faye et al., 2023). To ensure reliable and generalizable findings, this study diversified its sample by covering companies from various industries, regions, and organizational types across China. Using snowball sampling, over 120 companies were contacted, with nearly 100 selected based on their implementation of comprehensive reward strategies. These companies are located in more than 10 provinces in China and are widely distributed in 14 industry fields, such as information transmission, real estate, leasing, and business services. Knowledge-based employees born in the 1990s, working in roles such as management, R&D, and consulting, were surveyed. Initially, employees assessed their rewards, proactive personality, and demographics. A month later, supervisors evaluated their individual task and team member proactivity while employees continued assessing their collectivism traits and relationships with subordinates.

This study employs two methods—two time-point surveys and leader-rated proactivity assessments—to reduce common method bias. First, to control for the temporal effects on the implementation of TR strategies, we ensured a sufficient time interval between surveys. This approach minimizes respondents’ ability and motivation to recall their answers from previous surveys, helping maintain consistency when answering subsequent questions (Podsakoff et al., 2003; Podsakoff et al., 2024).

Drawing from research in management and organizational behavior that uses multiple time-point surveys, most studies typically have an interval of at least one week, with many using a two-week gap. However, considering the process between the implementation of TR strategies and employee behavioral responses—which likely includes policy communication, policy perception, and policy feedback—this study acknowledges that employees need time to respond to compensation policies. Additionally, by separating the survey on organizational compensation practices from the one on employees’ cultural orientation, we ensure that the two measures do not influence each other (Daniel and Zhan, 2023; Fan et al., 2022). However, to ensure a high completion rate for each survey, the time interval should not be too long. Therefore, a one-month gap was chosen between the two surveys to balance the need for sufficient time for response while maintaining a manageable interval for participant engagement (Carnevale et al., 2023). Then, the outcome variables for different proactive behaviors are assessed by leaders rather than through self-reports from employees. Research indicates that obtaining predictor variables from different sources helps prevent respondents’ mindsets or emotions from influencing their ratings of these variables (Podsakoff et al., 2024). Therefore, using leader-rated assessments allows for a more accurate reflection of employees’ actual performance. There is no concern about leader evaluations being biased, as the supervisor-subordinate relationship is controlled as a variable. However, to understand the true impact of the compensation practices implemented by the organization and whether employees’ perceptions align with the organization’s intended design, it is crucial to gather insights based on employees’ self-reports. Similarly, it may be difficult for leaders to accurately assess employees’ cultural orientations, as they may not always be aware of these traits. Hence, employee self-reports on total rewards and cultural orientations are clearly valuable in this context.

To address employee sensitivity to compensation policies, this study employed an anonymous survey method with sealed collection and confidentiality assurances to reduce social desirability bias. Each company designated a contact person, who was trained to guide participants and ensure proper data matching using unique identifiers for each employee and leader. Employees’ personal information was omitted from the surveys, and leader questionnaires only included the names of the employees being evaluated. Finally, only names matching the number provided by the contact were matched at the data analysis stage. This ensured anonymity while avoiding any potential bias in the match. To ensure data recovery quality and reward the hard work of contacts and participants, the research team promoted their enthusiasm by distributing small gifts on the spot or by mail. The study achieved an 84.85% response rate, successfully matching 336 valid responses. The survey used closed-ended items, randomized the question order, and allowed participants to withdraw at any time, ensuring anonymity. Maximum likelihood estimation was applied to handle missing data, effectively reducing nonresponse and common method biases (Newman, 2014). Among the sample, 45.2% of the enterprises were state-owned, 25.3% were large companies with over 500 employees, 59.2% were in their growth or maturity phases, and 33.3% were in a transition period. The socio-economic demographics of the respondents reveal that 44.9% of the employees were male, and 61.6% were born after 1995, with an average age of 29 years. All participants held at least a college degree, with 77.7% possessing a bachelor’s degree or higher. The average tenure with their company was 4 years, and the average duration of their working relationship with their leaders was 3 years.

### 3.2 Variables description

All questions in this study’s questionnaire were scored on a 6-point Likert scale, with 1 being “very non-compliant” and 6 being “very compliant.”

#### 3.2.1 Total rewards

The variable of TR was measured using a 33-item scale Jin and Yang (2022) and Zhou et al. (2024) tailored to the Chinese organizational context. This scale encompasses seven dimensions: pay security, pay equity, workload, work experience, employee care, career development, and perceived personal value. Examples of items include “The company provides me with sufficient room for development,” “Colleagues have a harmonious relationship and a cordial atmosphere,” “The company has established a clear and reasonable pay-for-performance system,” and “The company can guarantee rest on rest days and holidays.” The scale demonstrated robust psychometric properties, with a Cronbach’s alpha 0.937. Additionally, the average variance extracted (AVE) values for the seven dimensions were above 0.5, composite reliability (CR) values were above 0.7, and the correlation coefficients between dimensions were less than the square root of their respective AVE values, confirming the scale’s reliability and validity.

#### 3.2.2 Vertical versus horizontal collectivism

Vertical and Horizontal Collectivism were assessed using a widely utilized 4-item scale (Singelis et al., 1995). Sample items from the scale include “Respecting the team’s decisions is important to me” for vertical collectivism and “I would be proud if one of my coworkers were honored” for horizontal collectivism. The scale’s reliability was demonstrated with Cronbach’s alpha values of 0.845 for vertical and 0.863 for horizontal collectivism.

#### 3.2.3 Individual task proactivity vs. team member proactivity

Individual task and team member proactivity were evaluated using a 3-item scale (Griffin et al., 2017), a commonly employed scale for assessing maturity. For individual task proactivity, a sample item is “The employee will try to suggest ways to improve the completion of important work tasks.” For team member proactivity, representative items include “The employee will suggest ways to improve the team’s productivity” and “The employee suggests ways to improve the efficiency of the team’s work.” The scales demonstrated acceptable reliability, with Cronbach’s alpha values of 0.758 for individual task proactivity and 0.752 for team member proactivity, respectively.

#### 3.2.4 Control variables

In addition to basic demographic characteristics at the individual employee level, such as gender, age, and education, and job-related variables at the corporate level, such as tenure, rank, and title, this study includes two special control variables. These were selected due to their theoretical relevance to proactive behaviors and compensation practices. The first is the individual trait variable, proactive personality, which can significantly influence employee initiative. This inclusion is particularly relevant because the study also examines how two cultural orientation traits—vertical and horizontal collectivism—affect the relationship between compensation policies and employee proactive behavior. Extensive research has established that proactive personality is a key personality trait that drives proactive behavior (Parker et al., 2006; McCormick et al., 2019). Therefore, this study included proactive personality as a control variable, measured using the four items with the highest factor loadings scale (Bateman and Crant, 1993). Sample items include, “If I believe strongly in an idea, no difficulties can stop me from realizing it.” The scale’s reliability, indicated by Cronbach’s alpha, was 0.791. The study also controlled for the leader-employee relationship, as the outcome variable was assessed based on the leader’s evaluation. To account for this, the study controlled the duration of interactions and the quality of supervisor-subordinate relationships using a six-item scale (Law et al., 2000), which had a high Cronbach’s alpha of 0.871. Reliability was assessed using Cronbach’s alpha and composite reliability, all of which exceeded 0.700 (Mohi Ud Din and Zhang, 2023; Faye et al., 2025).

## 4 Results

### 4.1 Common method bias test and validation factor analysis

We first conducted Harman’s single-factor test using SPSS 26, which showed that the first factor explained 28.75% of the variance, well below the 50% threshold, indicating no significant common method bias (Podsakoff et al., 2003; Podsakoff et al., 2024). To address limitations in Harman’s test, we performed confirmatory factor analysis (CFA) using Mplus 8.3. The results revealed that the original five-factor model fit the data significantly better than alternative models. The fit indices for the five-factor model were: *χ*^2^ = 1903.96, df = 982, *χ*^2^/df = 1.939, RMSEA = 0.053, CFI = 0.909, TLI = 0.900, and SRMR = 0.062. The alternative models, including the one-factor, two-factor, three-factor, and four-factor models, showed poor fit, confirming that the five-factor model is the best fit for the data.

Additionally, adding an extra latent factor to the original five-factor model did not improve the fit, as the results were identical to those of the five-factor model. According to Mplus statistical analysis guidelines, an RMSEA value below 0.08 is considered acceptable, while the CFI and TLI values should exceed 0.9, and the SRMR should be close to 0.08 (Cheng et al., 2016). Only the original five-factor model met these criteria, with all alternative models showing poor fit. This indicates that it is valuable to study the two cultural orientations and two types of proactive behaviors separately. These results confirm strong discriminant validity among the individual internal variables (see Table 1).

**Table 1:** Validation factor analysis results.

Model	*χ* ^2^	df	*χ*^2^/df	RMSEA	CFI	TLI	SRMR
5-factor model + latent method factor	1903.960	982.000	1.939	0.053	0.909	0.900	0.062
5-factor model (TR, VC, HC, ITP, TMP)	1903.960	982.000	1.939	0.053	0.909	0.900	0.062
4-factor model (TR, VC, HC, ITP + TMP)	2058.46	986	2.088	0.057	0.894	0.884	0.064
4-factor model (TR, VC + HC, ITP, TMP)	2520.72	986	2.557	0.068	0.849	0.834	0.085
3-factor model (TR, VC + HC, ITP + TMP)	2666.17	989	2.696	0.071	0.834	0.819	0.086
2-factor model (TR, VC + HC + ITP + TMP)	2844.51	991	2.87	0.075	0.817	0.800	0.084
1-factor model (TR + VC + HC + ITP + TMP)	3229.28	992	3.255	0.082	0.779	0.759	0.080

*N* = 336; TR, total rewards; ITP, individual task proactivity; TMP, team member proactivity; VC, vertical collectivism; HC, horizontal collectivism; + denotes merging into one factor.

### 4.2 Descriptive statistics and correlation analysis

As indicated in Table 2, significant positive correlations were observed between TR and both individual task proactivity and team member proactivity, with correlation coefficients of 0.540 and 0.473, respectively, and both were statistically significant at the *p* < 0.01 level. Applying the dual criteria, the correlation coefficients are considerably below 0.75, and the variance inflation factors (VIF) for the core variables remain under 2—substantially below the commonly accepted threshold of 10. Therefore, this study exhibits no concerns of multicollinearity. These results preliminarily support the proposed hypotheses.

**Table 2:** Variable means, standard deviations, and correlation coefficients.

No.	Variables	Mean	SD	1	2	3	4	5
1.	Total rewards	4.597	0.753	1				
2.	Individual task proactivity	4.621	1.062	0.540^**^	1			
3.	Team member proactivity	4.703	0.993	0.473^**^	0.355^**^	1		
4.	Vertical collectivism	4.130	1.247	0.315^**^	0.274^**^	0.249^**^	1	
5.	Horizontal collectivism	4.154	1.320	0.381^**^	0.251^**^	0.410^**^	0.206^**^	1

### 4.3 Hypothesis testing

#### 4.3.1 Main effect

Although this study uses leader assessments to measure proactivity, the research focuses on individual-level variables, meaning there is no need to aggregate data at the team level. Therefore, a multilevel model was not chosen for this study. Instead, we opted to use hierarchical regression analysis in SPSS 26 to test the hypotheses. In Table 3, Models M1 and M4 test the main effects. The results indicate that TR significantly contributes to both individual task proactivity (*β* = 0.627, *p* < 0.001) and team member proactivity (*β* = 0.593, *p* < 0.001), supporting hypotheses H1a and H1b, respectively. The regression analysis reveals a more significant impact of TR on individual task proactivity (*β* = 0.627) than team member proactivity (*β* = 0.593). To determine whether this difference (*β* = 0.033) is statistically significant at the 0.05 level, the study follows established practices in the literature, calculating the 95% confidence intervals for both coefficients. A statistical difference is indicated if the intervals overlap by less than 50% (Tano et al., 2023). The analysis shows that the lower limit of the 95% confidence interval for the individual task proactivity coefficient is 0.594, and the upper limit is 0.861, while for the team member proactivity, the lower limit is 0.463, and the upper limit is 0.723. The overlap between these intervals does not exceed the 50% threshold, confirming that the difference is statistically significant and supporting hypothesis H1c.

**Table 3:** Hierarchical regression analysis results.

Variables	Individual task proactivity			Team member proactivity		
	M1	M2	M3	M4	M5	M6
Constants	1.122	1.408	0.958	1.145	1.025	1.759
Control variables						
Gender	0.089	0.101	0.078	−0.024	−0.033	−0.054
Age	−0.017	0.002	−0.018	−0.003	0.000	0.002
Time in office	0.013	−0.009	0.015	−0.017	−0.019	−0.012
Academic background	−0.034	−0.032	−0.028	0.016	0.024	0.021
Grade	−0.140	−0.157	−0.143	−0.013	−0.030	−0.071
Title	0.036	0.007	0.030	−0.036	−0.040	0.018
Co-working time	0.004	0.022	0.003	0.006	0.010	0.011
Enterprise nature	−0.044	−0.034	−0.033	−0.003	0.023	0.034
Regional background	0.010	0.012	0.008	0.001	0.000	0.012
Proactive personality	0.082	0.069	0.074	0.125^*^	0.116	0.089
Supervisor-subordinate relationship	0.099^*^	0.108	0.099	0.138^**^	0.131^*^	0.099
Dependent variable						
Total rewards	0.627^***^	0.536^***^	0.645^***^	0.593^***^	0.546^***^	0.308^***^
Moderating variables						
Vertical collectivism		0.052			0.073	
Horizontal collectivism			0.031			0.180^***^
Interaction term						
Total rewards^*^ vertical collectivism		−0.370^***^			−0.029	
Total rewards^*^ horizontal collectivism			0.057			−0.301^***^
*R* ^2^	0.315	0.426	0.318	0.260	0.268	0.391
Adj-*R*^2^	0.289	0.401	0.289	0.233	0.237	0.364
*F*	12.358^***^	17.033^***^	10.705^***^	9.462^***^	8.414^***^	14.717^***^

#### 4.3.2 Moderating effects test

This study used hierarchical stepwise regression in SPSS 26 to analyze the moderating roles of vertical and horizontal collectivist orientations. Independent and moderating variables were centered on reducing multicollinearity before constructing interaction terms for the regression analysis.

Table 3 shows the results of the four tests for moderating effects, with Models M2 and M5 specifically analyzing the impact of vertical collectivism. The interaction between TR and vertical collectivism was found to significantly reduce individual task proactivity (*β* = −0.370, *p* < 0.001), but it did not significantly affect team member proactivity (*β* = −0.029, n.s.). This suggests that as vertical collectivism increases, the positive relationship between TR and individual task proactivity weakens, while its effect on team member proactivity remains unchanged. Thus, hypothesis H2a is supported, while hypothesis H2b is not.

Models M3 and M6 tested the moderating effect of horizontal collectivism. The results in Table 3 show that the interaction between TR and horizontal collectivism was not significant for individual task proactivity (*β* = 0.057, n.s.) but had a significantly negative impact on team member proactivity (*β* = −0.301, *p* < 0.001). This suggests that as horizontal collectivism increases, the positive effect of TR on individual task proactivity remains unchanged, while its positive impact on team member proactivity diminishes. As a result, hypothesis H3a is not supported, whereas hypothesis H3b is confirmed.

**Figure 2:**
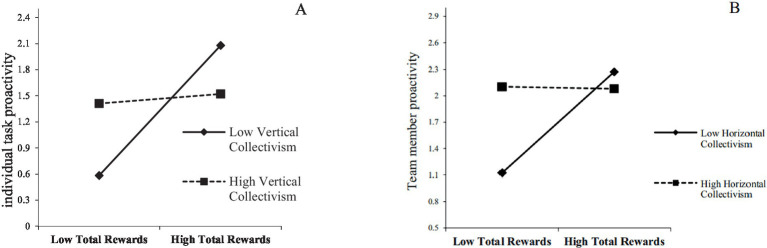
**(A)** H2a: The moderating effect of vertical collectivism on the relationship between total rewards and individual task proactivity. **(B)** H3b: The moderating effect of horizontal collectivism on the relationship between total rewards and team member proactivity.

Interaction plots were created using the mean plus or minus one standard deviation to illustrate these moderating effects better. Figure 2A reveals that the positive impact of TR on individual task proactivity is more robust among employees with low vertical collectivist orientations. Conversely, Figure 2B shows that as employees’ horizontal collectivism increases, the positive relationship between TR and team member proactivity weakens.

#### 4.3.3 Robustness tests

In this section, we will conduct robustness checks using two methods. The first method involves re-running the hierarchical regression analysis after removing the control variables to assess whether factors such as gender, age, academic background, tenure, regional background, and enterprise nature influence the results of this study. Similarly, the analysis was conducted using SPSS 26.0, and the results are presented in Table 4. The findings show that the main effects and moderation effects remain unchanged regardless of whether any control variables are included.

**Table 4:** Hierarchical regression analysis results (excluding control variables).

Variables	Personal task proactivity			Team member proactivity		
	M1	M2	M3	M4	M5	M6
Constant	1.120	1.855	0.884	1.838	1.734	2.575
Dependent variable						
Total reward	0.661^***^	0.566^***^	0.668^***^	0.623^***^	0.569^***^	0.312^***^
Moderating variable						
Vertical collectivism		0.066			0.087	
Horizontal collectivism			0.044			0.195^***^
Interaction term						
Total reward-vertical collectivism		−0.361^***^			−0.020	
Total reward-horizontal collectivism			0.059			−0.297^***^
*R* ^2^	0.292	0.405	0.297	0.223	0.235	0.368
Adj-*R*^2^	0.289	0.400	0.290	0.221	0.228	0.363
*F*	137.452^***^	75.428^***^	46.715^***^	96.074^***^	33.961^***^	64.538^***^

Additionally, this study also employed a substitution test method to assess the robustness of the model. Specifically, we employed Model 1 in the PROCESS macro (version 4.1) to re-examine the moderating effects (Hayes, 2018). The significance of the conditional process was assessed by checking whether the 95% confidence intervals for each moderating variable at different levels were obtained through the Bootstrap method (5,000 samples), including zero. The test results are presented in Table 5. The interaction term between TR and vertical collectivism includes zero in the test for team member proactivity, as does the interaction term between TR and horizontal collectivism in the test for individual task proactivity. The remaining interactions do not include zero.

**Table 5:** Robustness analysis using the PROCESS macro program.

	Variables	Individual task proactivity			Team member proactivity		
		Effect value	SE	95% confidence interval	Effect value	SE	95% confidence interval
Process level	Total rewards x vertical collectivism	−0.370	0.049	[−0.466, −0.274]	−0.029	0.052	[−0.130, 0.073]
	Total rewards x horizontal collectivism	0.060	0.052	[−0.045, 0.159]	−0.301	0.046	[−0.391–0.211]

Furthermore, we conducted a conditional effect analysis using the PROCESS macro, displaying the results for values one standard deviation above the mean, one standard deviation below the mean, and the inter-group differences, as shown in Table 6. The results indicate that when the value is one standard deviation below the mean, the 95% confidence interval does not include zero, whereas when it is one standard deviation above the mean, the 95% confidence interval includes zero. The inter-group differences do not include zero either. These findings provide further support for hypotheses H2a and H3b. These findings suggest that the moderating effects model constructed in this analysis exhibits strong robustness.

**Table 6:** Conditional effect analysis.

Process macro	Regulating variable	Outcome variable	Path	*B*	SE	95% confidence interval
M1	VC	ITP	Low (*−*1 SD)	1.232	0.097	[1.042, 1.422]
			Between-group variance	0.399	0.075	[0.251, 0.547]
			High (+1 SD)	0.121	0.099	[−0.073, 0.316]
	HC	TMP	Low (*−*1 SD)	0.88	0.087	[0.708, 1,052]
			Between-group variance	0.203	0.077	[0.052, 0.354]
			High (+1 SD)	−0.097	0.109	[−0.312, 0.118]

## 5 Discussion

This study explores how collectivist cultural orientations influence the effectiveness of compensation policies among knowledge workers. Findings show that TR boosts both individual task and team member proactivity, with a greater effect on individual task proactivity. However, vertical collectivism weakens the rewards-proactivity link for individuals, while horizontal collectivism weakens it for team member’s. The study confirms that vertical and horizontal collectivism are distinct, independent constructs (Triandis and Gelfand, 1998). Our study does not support the positive moderating effects of vertical collectivism on the relationship between TR and team member proactivity (H2b) or horizontal collectivism on individual task proactivity (H3a). This may be because vertical collectivism employees prioritize hierarchy and authority, reducing their willingness to challenge norms or engage in change (Triandis and Singelis, 1998). Similar findings were reported by Chiou and Pan (2008), who found higher vertical collectivism was associated with a high moral view.

Based on the social exchange theory, in a collective reward system like TR, reciprocity in terms of resources and behavior may only extend to compliance with existing rules and authority, making it unlikely to promote riskier behaviors such as team member initiative. On the other hand, if a member proposes workflow changes to improve team effectiveness and, after incurring the necessary costs, gains approval from the internal authority, other members may engage in “free-riding” behaviors, contributing less effort and reducing overall team initiative (Morris et al., 1994). The failure to support Hypothesis 3a may stem from the fact that individuals with strong horizontal collectivist tendencies may desire equality in expression but fear that suggesting changes in work practices could give the impression of trying to “stand out” or “show off,” potentially disrupting interpersonal harmony. Scholars suggest that individual proactive behaviors can sometimes affect colleagues’ tasks, and concerns about being expected to engage in social learning can reduce intrinsic motivation, even when the organization provides TR, thus lowering individual task initiative results (Ding et al., 2024). Zhang et al. (2022) provide evidence that performance-based pay enhances intrinsic motivation and creativity by increasing perceptions of autonomy. However, individuals with a high horizontal collectivist orientation who prioritize relationality over autonomy may experience a reduction in motivation from PFP.

The lack of significant findings can be explained by role conflict theory. Employees with strong vertical collectivism face conflicts between prioritizing collective goals and adhering to strict hierarchical norms, making it uncertain whether the compensation system enhances team member initiative. Similarly, employees with strong horizontal collectivism experience role conflict between maintaining harmony and advocating for equal expression, which makes it unclear whether the system promotes individual task initiative. Previous research shows role conflict leads to uncertainty (Anglin et al., 2022). However, for employees with low vertical or horizontal collectivism, role expectations align, strengthening individual or team initiative, making the compensation system more effective in enhancing initiative and improving performance.

### 5.1 Theoretical significance

This study examines how total compensation influences various forms of proactive behavior among the younger generation of Chinese knowledge employees while also exploring the moderating role of cultural orientation in this relationship. It provides valuable insights into organizational behavior and compensation practices within a cultural context. Additionally, the study investigates the underlying causes of the failure of compensation incentive policies to address workplace behaviors such as “lying flat” and “fishing” among China’s post-90s knowledge-based employees. By expanding research on incentives for this demographic, the study highlights the shifting competitive landscape in the knowledge and digital economies, where the attraction and retention of knowledge workers are paramount. As post-90s employees increasingly form the backbone of the workforce, their lack of individual or team proactivity risks wasting valuable human capital and undermining organizational competitiveness. Despite their significance, research on motivating this generation remains limited, making it a key area for future study.

Additionally, this study contributes to the understanding of proactive behavior by exploring the “black box” that differentiates between individual-oriented and team-oriented proactive behavior. While interest in proactive behaviors is growing, an insufficient exploration of their distinct classifications is vital in practical workplace settings. Some individuals may excel in personal tasks but falter in team settings, leading to issues like “social loafing.” This underscores the need to examine various proactive behaviors and their motivational drivers separately to enhance their effectiveness. Previous research has often focused on general or specific forms of proactive behavior, such as constructive behavior or job crafting. Still, there is a notable lack of studies that categorize different types of proactive behaviors by distinct goals, especially within a single investigation. Our study addresses this gap by examining the role of TR—an organizational pay policy—as an antecedent to various proactive behaviors (El Baroudi et al., 2019; Belschak and Den Hartog, 2010). Utilizing social exchange theory and role theory, we identify the boundary conditions influencing different proactive reciprocal behaviors, thereby contributing to a more comprehensive understanding of this field (Grant and Ashford, 2008; El Baroudi et al., 2019).

This study advances the empirical exploration of TR theory within Chinese organizational contexts, a concept that originated in western enterprises and was introduced to China after 2000. This study, focusing on the new generation of Chinese knowledge workers, confirms that TR effectively motivates proactive behaviors. It extends the applicability of this theory in the Chinese context, enhances understanding of its impact on different forms of proactive behavior, and helps bridge the gap between compensation theory and practice in China. Furthermore, while previous studies have typically examined single types of reward systems—such as performance pay or leadership recognition—in isolation, this study enriches the theoretical understanding of pay incentives and their consequences for proactive behaviors.

Additionally, this study makes a significant theoretical contribution by expanding the understanding of pay incentive effectiveness, incorporating individual differences in cultural value orientations. It explores how variations in collectivist cultural orientations influence the relationship between total rewards and proactive behaviors, providing empirical support for social exchange and role theories. The findings show that organizational support, rewards, and an individual’s collectivist orientation interact to shape proactive behaviors. Employees with strong horizontal collectivist tendencies may avoid behaviors that disrupt norms, even with organizational support, due to a focus on interpersonal harmony. The study also highlights regional cultural differences within China, where employees in eastern coastal regions, influenced by globalization, may prioritize equality and innovation, while those in western and rural regions may emphasize hierarchy and authority. Future research could explore how these regional differences affect the effectiveness of total rewards (TR) in motivating different proactive behaviors.

In contrast, those with a low vertical collectivist orientation are more likely to take self-directed initiatives, unconstrained by concerns about authority. Interestingly, the positive moderation of both collectivist orientations on proactive behaviors was not significant, possibly due to role conflict or the inherent nature of cultural values, which warrants further investigation in future research. While it is well-established that individual cultural differences significantly influence employees’ interpretation of various organizational behaviors, this study is the first to explore the boundary conditions between reward systems and proactive behaviors from the perspective of cultural heterogeneity. This study builds on the work of Mowbray et al. (2024), who suggested that theirs was the first empirical study to validate the relationship between rewards and voice behaviors. However, their research did not explore the boundary conditions that could enhance or attenuate the effectiveness of reward practices, nor did it theorize under what circumstances the impact of different rewards might vary. In this sense, the present study responds to their call for further investigation into these factors. Compensation policy, a crucial element for every company worldwide, is a key component of strategic human resource management. It plays a vital role in translating corporate strategies and objectives into successful outcomes (WorldatWork, 2024). In particular, the comprehensive total compensation design system is more developed and enduring in western contexts. The findings of this study offer valuable theoretical insights into the effectiveness of compensation policies implemented by corporate managers globally.

### 5.2 Practical implications

Companies should adopt a TR approach to motivation rather than relying solely on financial incentives or a limited set of non-financial rewards (Zhou et al., 2024). Our research highlights the critical role of TR in fostering proactive behaviors among young knowledge employees. A report by research firm Qualtrics on Global Employee Experience Trends 2024 found that 36% of non-frontline employees and 50% of frontline employees remain dissatisfied with their compensation packages, highlighting a significant gap between expectations and reality (Qualtrics, 2024). This suggests that designing an effective reward strategy is a complex task, especially in the context of the new normal of remote work and shifting employer expectations. Evolving workforce needs require more innovative and tailored approaches to compensation.

Organizations should go beyond market-based pay and develop comprehensive compensation strategies that include health insurance, wellness support, retirement plans, and non-financial incentives like meaningful work, leadership recognition, career advancement, and flexible work options. Effective communication and understanding employee needs are essential, as different groups perceive valuable compensation differently. Tailoring compensation strategies to employees’ cultural orientations and regional contexts is crucial. For example, the effectiveness of rewards for task proactivity can be improved by reducing levels in areas with high levels of vertical collectivism, such as rural areas. Enhancing the effectiveness of rewards in improving team member proactivity by reducing areas with high levels of horizontal collectivism, such as coastal areas. By adapting compensation strategies to cultural values and regional differences, organizations can motivate proactive behaviors more effectively and ensure that their approaches are culturally sensitive.

Furthermore, as companies increasingly adopt data analytics and artificial intelligence to align HR practices with employee attitudes and behaviors, managers can leverage data-driven technologies to optimize TR strategies, ensuring efficient use of resources and better alignment with employee needs. For example, Bayer, a well-established European company, introduced a digital incentive platform—the “Bayer China Employee Recognition Program”—which leverages cloud computing, big data, mobile, and social technologies. This platform enhances employee engagement by fostering real-time recognition and communication between management and employees, as well as among employees themselves. It features dynamic scenarios and digital medals to acknowledge contributions, which significantly improves the employee experience. The program’s impact is evident: the average time spent per user per visit was 23 min, with an average of four “thank you” cards sent per user each month. Users visited 89 pages, performing 43 actions per visit. Following the program’s implementation, employee motivation, satisfaction, and dedication increased, leading to improved talent retention and attraction (CDP Dynamics, 2019).

However, when implementing TR, organizations must understand the vertical and horizontal collectivist values of their employees and assess behaviors such as “lying flat” or “fishing.” This understanding allows organizations to develop tailored strategies to address resistance to change, whether it originates at the individual or team level. There are two key strategies: organizational interventions at the macro level and personalized improvements at the micro level.

At the macro level, if an organization finds widespread poor individual task initiative, it may benefit from fostering a non-authoritarian, non-hierarchical, and non-competitive corporate culture to mitigate the effects of vertical collectivism. Additionally, adjusting recruitment and selection processes to reduce the hiring of employees with strong vertical collectivist tendencies can enhance the effectiveness of compensation policies.

On the other hand, if an organization faces widespread social loafing, where employees are unwilling to contribute their knowledge and skills to the team, promoting a culture focused on equality and harmony could backfire. In such cases, a different approach is needed to address these challenges.

At the micro level, managers should use personalized strategies to address employee disengagement. For employees exhibiting “lazy” behavior, it is crucial to stimulate intrinsic motivation by recognizing effort, not just results. Acknowledging their contributions can help reduce the influence of vertical collectivism. In contrast, for team members inclined to “unite,” managers should focus on fostering a sense of authority and encouraging a competitive spirit to counterbalance their horizontal collectivist tendencies. This can also be integrated into the organization’s overall compensation strategy. For example, to cultivate a harmonious and egalitarian corporate culture, a program similar to Bayer China’s Employee Recognition Program could be implemented. This might involve setting up thank-you cards between different departments to foster a collaborative atmosphere genuinely. On the other hand, to encourage a competitive spirit, the TR model could include mechanisms such as promotions, role-model learning, and PFP.

### 5.3 Limitations and prospects

This study has a few limitations that should be acknowledged and which suggest directions for future research. First, although the study employed a two-time point, paired questionnaire survey design to minimize common methodological bias, the one-month interval between surveys still renders the study cross-sectional. Future research could adopt a longitudinal design to better clarify causal relationships. For instance, changes in employees’ proactive behaviors could be assessed after 3 months, 6 months, or a year to explore the long-term effects of TR implementation. Experimental methods could also be employed to examine whether the participants’ collectivist orientation influences their proactive behavior, testing their mental state in relation to initiative performance.

Second, this study focused on exploring the reasons for the failure of pay policies from the perspective of intra-cultural heterogeneity. However, it did not examine the intrinsic mechanisms linking TR to different initiative forms, such as potential mediating factors. Future research could leverage theories such as social exchange theory and proactive motivation models to explore these mechanisms further. Additionally, it could examine whether cultural orientation still moderates the relationship when mediation effects are present, thereby enhancing the contribution of the study.

Third, this study does not account for regional differences in cultural values within China. Future research could explore how regional cultural nuances, such as the prevalence of vertical collectivism (VC) in western regions or horizontal collectivism (HC) in eastern regions, influence the effectiveness of total rewards (TR) in motivating proactive behaviors. Such regional analyses would provide a more nuanced understanding of cultural influences on compensation practices.

Additionally, future studies could logically derive and empirically validate the impact of other cultural value orientations on these relationships. Fourth, our findings may be generalizable to other collectivist national contexts since cultural orientations exist in all countries. Additionally, these results could be extended to different organizational settings and other employee groups across various countries. Finally, future research could explore other cultural factors (such as power distance, risk aversion, etc.) or organizational factors that may influence the relationship between TR and proactive behavior.

## 6 Conclusion

This study investigates the boundary conditions of the impact of total rewards (TR) on two distinct forms of proactive behavior: individual task proactivity (ITP) and team member proactivity (TMP). By integrating social exchange theory and role theory and focusing on the new generation of Chinese knowledge employees, we find that TR significantly enhances both types of proactive behavior, with a more significant positive effect on ITP than on TMP. However, vertical collectivist orientation (VC) weakens the relationship between TR and ITP, while horizontal collectivist orientation (HC) weakens the relationship between TR and TMP. This study sheds light on why and under what conditions organizational pay practices may fail, viewed through the lens of intra-cultural heterogeneity. It also contributes to the theoretical understanding of TR and different initiative forms. It offers valuable insights for managers seeking to mobilize various forms of initiative among the new generation of knowledgeable employees.

## Data Availability

The raw data supporting the conclusions of this article will be made available by the authors, without undue reservation.
